# Engaging me softly: Comparing social drivers for continuative citizens’ participation in a long-term citizen science initiative on protected species monitoring

**DOI:** 10.1371/journal.pone.0324701

**Published:** 2025-06-10

**Authors:** Silvia Gisondi, Paolo Giardullo, Alice Lenzi, Emanuele Di Buccio, Pio Federico Roversi, Alessandro Campanaro

**Affiliations:** 1 CREA – Research centre for Plant Protection and Certification, Rome, Italy; 2 Department of Philosophy, Sociology, Education and Applied Psychology, University of Padova, Padua, Italy; 3 CREA – Research centre for Plant Protection and Certification, Florence, Italy; 4 Department of Life Sciences, University of Siena, Siena, Italy; 5 Department of Information Engineering, University of Padova, Padua, Italy; 6 Department of Statistical Sciences, University of Padova, Padua, Italy; Saint Xavier's College, INDIA

## Abstract

The engagement of volunteers in Citizen Science (CS) projects is a relevant issue that needs to be addressed to ensure long-term sustainability, scientific relevance, and public participation. Given this, the present paper analyses the social drivers of volunteers’ involvement in a long-term CS initiative to monitor protected species and habitats all over Italian national territory. This initiative was initially born as the LIFE11 NAT/IT/000252 MIPP (Monitoring of Insects with Public Participation), then changed name to “InNat” benefitting from Italian national fundings, and it finally ended in 2024. Overall, it counts more than 1600 participants to whom a dedicated survey was submitted in 2022 to compare and analyse different factors potentially driving participation (potential enablers) and fostering engagement within the project. Based on the survey results (22.3% response rate of 1632 invitations sent), different drivers for participation are modelled (socio-demographic features, interest in scientific topics, environmental attitudes) considering the following main factors: (i) the level of commitment to the initiative, (ii) the seniority of the citizen scientist involved, (iii) the attitudes towards nature conservation and species monitoring, (iv) the value assigned to CS activities. In this context, socio-demographic variables have been compared to attitudes and practices connected to open-air monitoring activities (e.g., recording protected species and habitats by taking pictures in nature). The proposed analyses tackle a variety of cultural and social components as well as their relationship, highlighting some of the features (e.g., active interest in CS activities reverberating in both commitments to engage other volunteers and active search for CS initiatives) that characterize constant participation. We classified volunteers into two categories (i.e., Consistent Volunteers and Non-Consistent Volunteers), comparing these two categories along potential enablers of engagement. Results show homogeneity among volunteers for several parameters (e.g., similar education level, age, occupational status) but also differences in personal motivation and active interest in citizen science initiatives.

## 1. Introduction

The engagement of volunteers in Citizen Science (CS) projects is a relevant issue to be addressed in order to ensure long- term sustainability, scientific relevance and public participation. Given this, the present paper analyses the social drivers of volunteers participation in a long-term CS initiative aimed at monitoring protected species and habitats all over Italian national territory. In the last decades, scientists have been facing an alarming scenario of biodiversity decline and habitat fragmentation [1,2], resulting in the loss of both species diversity and biomass. In particular, insects are affected by this decline, especially concerning their abundance

[3–6]. At the same time, several red lists and ‘protection lists’ targeting insect species are flourishing in order to identify the most vulnerable and endangered taxa (saproxylic beetles: [7]; grasshoppers, crickets and bush-crickets: [8]; dragonflies: [9] ; bees: [10]; butterflies: [11]; Mediterranean butterflies: [12]). Given this context, from the perspective of conservation biology, any data supporting scientists to obtain information on species distribution at various spatial scales and to monitor changes in species presence over time prove to be invaluable. Such data allow for the assessment of population and species conservation status, the identification of pressures and threats, and subsequently the implementation of effective management and conservation strategies. Indeed, the European Commission mandates that member States conduct surveillance of biodiversity through continuous monitoring (article 11 of the Habitats Directive 94/43/ECC) of protected species and habitats (i.e., listed in Annexes I, II and IV of the Habitats Directive 94/43/ECC) and provide regular reporting every 6 years (ex Art. 17 of Habitats Directive 94/43/ECC). However, presence data for species at national scale is challenging to obtain and requires a substantial sampling effort in terms of time allocated and area covered, especially for insects. Such conditions result in significant challenges for both scientists and managers of protected areas, so that 80% of the above-mentioned monitoring activities are implemented and carried out thanks to the involvement of volunteers [13; 14], adopting a CS approach. CS is commonly recognised as a collaborative method for developing scientific understanding. This approach effectively helps bridging the gap between specialists and non-experts [15], which is considered a key obstacle in contemporary scientific governance [16]. CS also constitutes a relevant method [17] especially when applied to environmental monitoring [18], as also highlighted by the frequent overlap of the keyword “citizen science” with the keywords “monitoring”, “climate change” and “invasive species” [19]. As a matter of fact, up to date, there are a number of CS projects that engage the public in studying and monitoring biodiversity. Concerning insects, such initiatives mostly focus on well-detectable and “charismatic” species with key ecological roles, such as pollinators [20,21], easily findable/identifiable taxa such as butterflies [22] or large and charismatic saproxylic beetles [23,24; 25]. In addition, CS is gaining its momentum in many fields also thanks to the development and diffusion of digital technologies; according to previous work [26], Information Communication Technologies (ICTs) are expected to create opportunities for large participation of a wide array of non-expert. Many organisations developed CS projects based on mobile applications and web-apps connected to large web platforms: among all of these, iNaturalist (inaturalist.org) and Zooniverse (zooniverse.org) are the most famous, but other recently developed initiatives are keen to explore ICTs opportunities [27]. Likewise, communication campaigns of projects, aimed at recruiting participants, also tend to follow online strategies [28,29]. The monitoring of protected species seems to be a promising domain of application for CS as a method of public engagement and awareness raising: CS provides an opportunity to foster biodiversity and the monitoring of protected species [30–32] while it actively engages members of the public in cutting-edge scientific research questions, in the attempt to reduce the existing gap between science and society [18,27,29,33,34].

However, three relevant challenges concerning the application of CS to environmental monitoring and biodiversity conservation are: i. the public awareness on biodiversity-related topics, ii. the long-term commitment of the participants and iii. the standardisation of data gathering [35,36]. The way these three challenges are addressed can severely affect the success or the failure and termination of a CS project [26], thus resulting in a lack of biodiversity data for conservation purposes. Firstly, although public awareness of biodiversity loss is certainly growing [37], the ecological relationships between different and less known domains of the biosphere (e.g., neglected species as insects, or arthropods more in general) are harder to grasp for non-professional scientists [38]. Then, the retention of participants, though receiving comparatively little attention as a topic [39], is extremely relevant for CS applied to environmental monitoring since such projects have specific features, such as territorial extension and diachronic analyses [40,41], that can amplify the need to retain participants, keeping their motivation up with both self-directed and altruistic incentives. Retention of participants can offer a number of benefits for the project itself, ranging from saving time on recruiting and training new participants, to producing more reliable observation records for documenting site changes over time. Moreover, long-term engaged volunteers may have a greater chance of being satisfied by their activity, developing a more profound connection to the project and ensuring greater willingness and reliability in this context. Finally, data standardisation and comparability play a crucial role in the interoperability of different data sources for ecological analyses at national and international spatial resolution, therefore this represents a major challenge for all CS projects focusing on different taxa.

In this context, an example of CS applied to conservation strategies can be found in the initiative ‘InNat’: born in 2012 as the LIFE11 NAT/IT/000252 “MIPP/InNat initiative” project, it changed name in InNat in 2017 under Italian national fundings and then it ended in February 2024. The aim of this initiative was to involve citizens in the collection of presence data of protected habitats and species all over Italy. Originally the project started targeting nine protected species (five saproxylic beetles, three butterflies and one bush cricket) under the LIFE MIPP project [42], but during the years target species increased in number and, in the end, InNat focused on 40 targets including eight dragonflies, two orthoptera, seven beetles, 17 butterflies, one freshwater crustacean, three plant species and two habitats. All the above-mentioned targets are protected under the Annexes I, II and IV of the Habitats Directive. Given the different funding resources as well as the wide temporal span of the initiative, volunteers recruitment followed a number of strategies which included: organization of promotional events, dedicated lessons at school, workshops/seminars at university, use of mass media (i.e., articles in newspapers and magazines, participation to television shows and radio broadcasts, dedicated documentary) and social media (i.e., Facebook, Instagram, Twitter), project website and newsletter. In respect to other platforms for the collection of biodiversity data (e.g., iNaturalist), the one developed by MIPP/InNat initiative (https://lifemipp.eu/ or the smartphone app ‘InNat’) relies on two main factors: i. data collection is required only for target species, i.e., citizens are actively motivated to recognize the target species and take pictures of those; ii. every geo- located picture uploaded on the platform by volunteers is validated by the expert staff prior to its inclusion in the project database. Data collected during MIPP/InNat initiative is publicly available on Global Biodiversity Information Facility (GBIF) repository [43]. The present paper analyses the results of a dedicated survey proposed to the participants of the above-mentioned long-term CS initiative, exploring factors that may potentially drive participation, fostering engagement within the project and eventually contributing to participation in a CS project with continuity over time. We call these factors “*potential enablers”* of engagement to volunteer into the project. The aim is to assess the features that may drive a participant to be engaged across time. Moreover, the analyses address some of the central issues of CS as reported above, such as: commitment to a CS project, sustainability of a scientific research project based on volunteers’ contributions, and desired and desirable effects of CS initiatives, such as the mobilisation and involvement of people who are usually outside the processes of scientific knowledge construction. Based on the survey results, different potential sets of drivers for participation have been investigated into detail. In this context, socio-demographic variables have been compared to attitudes and practices connected to open-air monitoring activities (i.e., recording protected species and habitats by taking pictures in nature). Data have been analysed along five areas, namely: i. socio-demographic features of participants (e.g., age class, education attainment); ii. interest in scientific topics (to what extent volunteers are interested into scientific issues in general and biodiversity more specifically); iii. personal attitudes related to the environment (ecocentrism and interest in conservation); iv. to what extent CS activities are part of volunteers’ leisure time; v. communication strategies through digital technologies. Each area has been reconstructed considering 15 variables reported in the literature as crucial to predict engagement into CS projects.

## 2. Methods

An online questionnaire, administered online through Limesurvey [44], was proposed to the volunteers who took part in the MIPP/InNat initiative in the period 2012−2021 and registered to the InNat portal. Participants received personalised contact emails, inviting them to take part in the survey. The web survey incorporated the subject heading “Survey InNat” in the email. Participants were informed that participation was voluntary and provided instructions to contact the research team for any inquiries or additional information. They were also informed that those interested would receive a brief summary of the study results. The confidentiality of responses was guaranteed. The response rate was calculated by dividing the number of returned questionnaires by the total number of questionnaires that reached potential respondents. The questionnaire comprised six sections and a total of 36 questions (see supplementary materials S1). The survey collected socio-demographic data about volunteers (section 1) and opinions on their role as volunteers in a citizen science project (section 2). In the latter section, two more elements have been added, namely those related to the historical contingency in which data was collected and a specific slant related to communication. Regarding the former element, it should be noted that data was collected in February 2022: at that time there were still many restrictions on personal mobility in Italy due to the Covid-19 pandemic and, at best, some restrictions were gradually being lessened [45]. For this reason, we included some questions on the extent of the lockdown. Then, the latter element about communication channels was investigated testing the impact of a) MIPP/InNat’s initiative promotion tools (e.g., public events; open lectures; web advertising); b) users’ preferred mode of uploading records. Subsequently, the survey collected information about any possible previous CS experience (section 3), it performed a test on the acquired skills on the monitored insect species (section 4), and it recorded opinions concerning the ecological crisis, the level of awareness of humankind-environment ecological relationship, and the trust in the ability of humankind to rule the nature and even to solve environmental issues, respectively (section 5): in the last section, ecocentric and promethean values were measured using the New Ecological Paradigm (NEP) scale [46]. A reduced NEP scale was employed, with eight statements out of the original 15, to which respondents are asked to express their level of agreement on a four-degree scale. The eight statements are roughly divided in two subgroups: one group reverbs ecocentric values (i.e., awareness of ecological crisis, its relevance and human responsibility) and the second group gathers items for promethean values (i.e., trust in human progress, technological solutionism). Finally, each respondent was asked to agree to being further contacted for possible interviews (section 6). The questionnaire as well as the anonymized responses are publicly available on Zenodo repository [47].

The survey respondents contributed a total of 3,709 reports, constituting 47.9% of the total reports submitted to the MIPP/InNat.

As reported in Table 1, the data show that survey respondents exhibit, on average, a higher level of engagement compared to the overall cohort of volunteers who submitted at least one report to the MIPP/InNat project. The former cohort exhibited a mean of 10 submissions per participant, in contrast to 4 for the latter. Furthermore, the data reveal that a minimum of 25% of survey respondents contributed no fewer than 6 reports, suggesting a notably higher level of participation.

**Table 1:** Comparison between total MIPP/InNat Volunteers and survey respondents: descriptive statistics about reports submitted.

	MIPP/InNat	Survey respondents
Volunteers	1632	364
Reports per volunteer		
Mean	4	10
Dev Std	21	36
Min	1	1
1st quartile	1	1
Median	1	2
3rd quartile	3	6
Max	572	437
Tot	7,738	3,709

In order to perform the analysis, we identified two classes of participants according to their “continuity” in the activity of reporting species. In the remainder of this paper, we will name the two classes as CV (Consistent Volunteers) and NVC (Non-consistent Volunteers). To split the volunteers in the two classes, we defined a Regularity Index (RI). The index considers both seniority and the number of reports sent from the first year of reporting up to 2021. Seniority is the number of years since the first confirmed record reported by a participant. The RI is defined as follows:


RI = i / max_i

where “i” is defined as follows:

i = (total number of records sent per participant)/ (seniority in years)

The value of “i” is normalised using max_i, i.e., the maximum value of i computed over all the volunteers. The basic idea underlying the index is to consider a combination of these two elements, i.e., “seniority” and “total number of reports”. The allocation of responding volunteers into the classes was based on the value of the Regularity Index (RI) and a threshold. The selection of the threshold was performed in order to include in Class CV those volunteers that provided at least two reports per year, looking at the average of all the years. However, we did not want to consider only this criterion to divide the volunteers in the two classes. In fact, our intent was to put in Class CV those volunteers: (a) engaged for a high number of years; (b) with at least a certain average of reports per year (approximately 2); (c) with a high number of reports. Therefore, we manually labelled all the 364 volunteers either as “consistent” or “not consistent” according to (b). Then we varied the value of the threshold from 0.001 to 1 and looked that the capability of the threshold in terms of maximizing the F1 measure, the number of years and the total number of reports. The F1 measure was computed as


F1 = TP / (TP + (FP + FN)/2)

where:

- TP is the number of volunteers labelled as consistent and correctly allocated in Class CV
- FP is the number of volunteers labelled as non-consistent but allocated in Class CV
- FN is the number of volunteers labelled as consistent but allocated in Class NCV.

As shown in Table 1, the highest value of F1 was obtained with a threshold value of 0.016; however, we were not only interested in “maximizing” F1, but we also wanted to consider the high number of years (seniority) and the high number of reports. All of these led to the selection of 0.018 as a threshold. In fact, in our case, the “optimal” threshold is not the one that maximizes only criterion (b) – used for manual labelling – but the one that satisfies all the three criteria (a-c) as much as possible. We carried out the same analysis by labelling as “non-consistent” the volunteers who satisfied the criterion of the average number of reports per year (b), but who also provided their records in a short time span (e.g., 1 year). The procedure led to the same threshold value. We opted to use the threshold to have the possibility to automatically process a high number of participants, if necessary for future works. This given, Consistent Volunteers (CV) class comprises volunteers with RI>=0.018, while Non-Consistent Volunteers (NCV) class comprises volunteers with RI < 0.018.

Results were analysed using Jamovi Version 2.3 [48], a free and open software based on a statistical spreadsheet built on top of the R statistical language [49]. To explore the relationship between variables, we performed a Principal Component Analysis (PCA, varimax rotation) (see supplementary materials). The variables are connected along eight factors that partially align with potential enablers recorded in literature. It should be noted that PCA has shown the connection between variables: this offered further robustness for the construction of the thematic areas in our analysis. We tested a binomial regression model considering the CV and NCV classes as dependent variable: the model proved to be inefficient (R²McF = 0.146, Confidence Interval 95%) and most of the variables resulted not relevant in predicting the belonging to a class of consistent or to a class of non-consistent volunteers (see supplementary materials S2). Consequently, data have been analysed using contingency analysis taking into consideration five areas encompassing the 15 variables reported in the literature as crucial to predict engagement into CS projects (Table 3). Given that most of the variable are ordinal we largely used Kendall’s Tau-b; it is a correlation coefficient that measures the strength and direction of association that exists between two variables measured on at least an ordinal scale. A risk analysis was also performed to assess differences in the sociability between the two identified classes and a selection of subgroups (e.g., age, gender). The risk analysis is based on a squared table 2x2. Odds ratio (OR) serves as a metric to gauge the relationship between a specific variable and its subsequent effect on two different groups: it essentially quantifies the likelihood of the occurrence of an outcome based on a given factor, relative to the likelihood of the occurrence of the same outcome in the absence of that factor. Relative Risk (RR) is a measure of the risk of a certain event happening in one group compared to the risk of the same event happening in another group.

**Table 2:** Performance of RI’s values in classifying respondents. The first column reports the value of the threshold, the second column reports the F1 value, the third column represents the average number of years in Class CV, and the fourth column refers to the average number of total reports computed over all the volunteers in Class CV. The underlined values show the thresholds established.

RI	F1	Average years	Average number of reports
0.010	0.69	3.67	18.96
0.011	0.89	4.47	27.41
0.012	0.89	4.47	27.41
0.013	0.92	4.48	28.79
0.014	0.94	4.48	29.64
0.015	0.94	4.45	29.83
0.016	1.00	4.56	32.95
0.017	0.98	4.53	34.02
0.018	0.94	4.46	35.77
0.019	0.94	4.46	35.77
0.020	0.94	4.46	35.77
0.021	0.85	4.94	42.57
0.022	0.83	4.87	43.36
0.023	0.82	4.86	43.85
0.024	0.81	4.86	44.86
0.025	0.81	4.86	44.86

**Table 3:** The five areas summarizing potential enablers recorded through 15 variables considered for the present paper. A general overview of the results is provided, as well as the main differences identified between the two classes of participants (n = 364), i.e., class CV – Consistent Volunteers (n = 94) and class NCV – Non-Consistent Volunteers (n = 270).

Potential enablers	Variables	General overview	Differences between Classes
1 – Socio-demographic features of participants	Age	Mainly mature participants: age average: 48.2 y.o.	No statistically significant differences between classes (ANOVA regression F = 0.182; p = 0.670)
	Gender	Male: 61.5% Female: 37.9% Other: 0.6%	No statistically significant differences between classes (X^2^ = 0.743 p = 0.690)
	Education	High level of education (degree or more): 58%	No statistically significant differences between classes (X^2^ = 4.82 p = 0.850)
	Working condition	Occupied: 60% high school teachers: 8.2% researchers or academics: 4.1%	No statistically significant differences between classes (X^2^ = 1.20 p = 0.878)
	Residency	Urban area: 52%	No statistically significant differences between classes (X^2^ = 2.39 p = 0.664)
2 – Interest in scientific topic	Passion for entomology	Passionate for bugs: 31.5%	No statistically significant differences between classes (X^2^ = 1.64 p = 0.650)
	Fascination in natural science	Fascinated by natural sciences: 31.8%	No statistically significant differences between classes (X^2^ = 2.01 p = 0.570)
	Usage of data for research reason	Use data from the LIFE MIPP/InNat initiative for research purposes: 31%	No statistically significant differences between classes (X^2^ = 5.09 p = 0.166)
3 – Ecocentrism and conservation	Ecocentrism/ prometheanism	Ecocentric values: 56% Promethean values: 12.3%	Similar values between classes but index associated differently between them (see Table 4)
	Feeling useful to nature conservation	Feel useful to nature conservation: 87.6%	Class CV: 90% are more likely to feel useful to nature conservation; class NCV participant: 86% are more likely to feel useful to nature conservation (X^2^ = 8.13 p = 0.043)
	Feeling closer to nature	Feel closer to nature in taking part to the project: 85%	No statistically significant differences between classes (X^2^ = 3.08 p = 0.379)
4 – Leisure time	Where and when people signal	During their free time: 82.1% During dedicated events: 5.9%	Class CV participants: 74.5% collect data in dedicated excursions Class NCV participant: 56.7% collect data in dedicated excursions (X^2^ = 11.4 p = 0.003)
	Sociability	Inviting other people to MIPP/InNat activities: 66%	Class CV participants tend to involve other people, mostly male aged 31–45 years (see Table 5)
	Covid Effect	Having suffered for not collecting data during the Covid-19 pandemic lockdown: 47.5%	Class CV participants: 66.6% Class NCV participants: 41.1% (see Table 6)
5 – Communication and use of digital technologies	Preferred channel for sharing data	Preferential use of the app: 66.8%	No statistically significant differences between classes (X^2^ = 0.364 p = 0.948)
	How participants learned about the project	Meeting the project leaders: 36% Querying on web: 26% By word of mouth: 26.6%	Class CV participants: 33.3% learned about the project autonomously (e.g., querying) on the web (see Table 7).

## 3. Results

Out of a total of 1632 volunteers, 364 completed the questionnaire, resulting in a success rate of 22.3%. Responding volunteers were divided in two classes based on the above defined regularity index (RI) and the identified threshold value (RI = 0.018): Consistent Volunteers (CV) class comprises 94 volunteers, while Non-Consistent Volunteers (NCV) class comprises 270 volunteers.

Since the RI might not fully capture some phenomena, such as an initial peak in engagement and a significant decrease over time, for volunteers in Class CV we visually inspected the presence of a possible decrease of engagement over time; in this case the engagement was measured in terms of reports per year. Within volunteers engaged for at least three years (60/94), 26.6% showed a negative slope in the regression line; however, only 15% contributed with one report in the last year of activity. Results from the two classes were compared along potential enablers of engagement recorded through variables selected from literature (see Table 2). Results will be commented throughout this section checking sets of variables related to the following potential enablers: i) Socio-demographic features of participants, ii) Interest in scientific topics, iii) Ecocentrism and conservation, iv) Leisure time, and v) Communication and use of digital technologies.

### 3.1. Socio-demographic features

A first set of potential enablers consists of socio-demographic features such as age, educational attainment, gender, residency and working condition. Respondents were quite homogeneous considering the different variables we recorded (Table 2). MIPP/InNat involved mature people (average = 48 years old), predominantly male (61% of the total), with a high level of education: 44% own a university degree and this percentage is about 20% higher compared to the share of Italian population holding a degree (23.1% on population between 25 and 64 years old). Within this context, Class NCV results to be slightly younger than Class CV, but the difference is not statistically significant (p-value = 0.809). The two Classes confirm the same general composition in terms of working conditions: volunteers are predominantly made up of employees (77% of the total of respondents) with a few researchers or university teachers (11.6%). From a territorial point of view, the distribution of volunteers results to be similar throughout the country, with a clear predominance of volunteers living in small and medium-sized towns (more than 60% of the total) and in Northern or Central Italy. These data are in line with national statistics. We noticed that there is a slight over-representation (+ 4%) of respondents living in rural areas: 44% vs the 48% recorded nationally by official data. This proportion does not change significantly between the two classes.

### 3.2. Interest in scientific topics

Considering the interest in scientific topics as the second potential enabler for engagement, the two classes show a general interest in scientific topics and more specifically entomology: 31.5% of respondents admit being passionate about insects while 31.8% declare to be interested in natural sciences. Given this, 31.1% of the respondents are considering using the project data for research purposes and 33.0% consider MIPP/InNat data as a useful tool to identify suitable sites where they may observe or photograph the target species (instrumental interest) with a barely significant difference between classes, respectively 27.7% for CV and 34.8% for NCV (X^2^ = 7.40; p = 0.060) and a weak association between classes and instrumental interest (Tau-b = 0.120; p = 0.013). Previous experience in CS projects does not seem to be decisive, and no significant differences can be found in the two classes (X^2^ = 0.172; p = 0.678). However, 42.9% of respondents stated to have already had experience in CS projects before getting involved in MIPP/InNat initiative.

### 3.3. Ecocentrism and conservation

**Fig 1:**
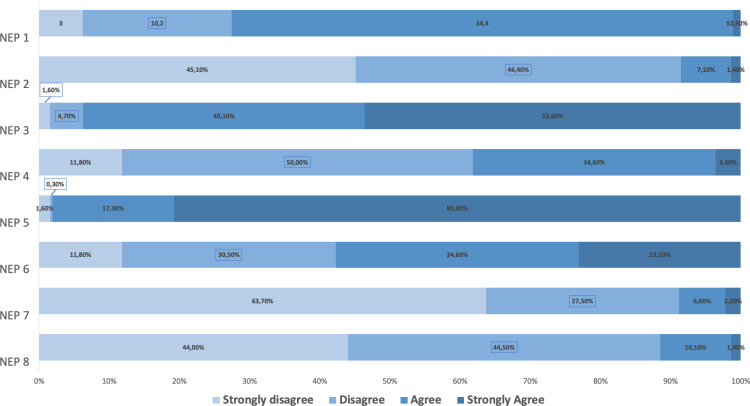
New Ecological Paradigm (NEP) Scale analysis: agreement and disagreement levels (i.e., strongly disagree, disagree, agree and strongly agree) about eight selected NEP Items. NEP1: we are approaching the limit of the number of people the earth can support (Ecocentrism); NEP2: humans have the right to modify the natural environment to suit their needs (Prometheanism); NEP3: if humans interfere with nature it often produces disastrous consequences (Ecocentrism); NEP4: human ingenuity will insure that we do not make the Earth unlivable (Prometheanism); NEP5: humans are seriously abusing the environment (Ecocentrism); NEP6: the Earth has plenty of natural resources if we just learn how to use them (Prometheanism); NEP7: the so-called “ecological crisis” facing humankind has been greatly exaggerated (Prometheanism); NEP8: humans will eventually learn enough about how nature works to be able to control it (Prometheanism).

The two classes share the attitude concerning outdoor activities: 84.4% of volunteers feel to be closer to nature when collecting data for MIPP/InNat and 87.7% feel that their involvement in MIPP/InNat is useful for nature conservation. Then, these feelings match with volunteers sharing a strong feeling on the urgency of ecological crisis: the majority of respondents reports strong agreement concerning statements outlining awareness of the ecological crisis and the relative human responsibility (Fig 1).

Looking at the distribution of responses, we can notice high level of agreement for items that can be easily interpreted as Ecocentric (NEP 1; NEP 3; NEP 5; NEP 7). However, we also noticed some trust attributed to human ability in mitigating ecological crisis (NEP 4) and in extracting resources from the environment (NEP 6). We used the items of the NEP scale to build two indexes and make comparisons between the two classes to measure the inclination of respondents both towards prometheanism and another towards ecocentrism. A joint analysis of the classes of indexes’ score derived from these items (Table 4) shows that ecocentric values are negatively associated with promethean values (Kendall’s Tau-b: −0.236; p < 0.001).

**Table 4:** Contingency analysis of promethean and ecocentric attitudes in classes: comparison between participants.

				Ecocentrism classes			Total
Participant Classes	Kendall’s Tau-b	p-value	Prometheanism classes	Low	Average	High	
**Class CV – Consistent participants (n = 94)**	**−0.443**	**< 0.001**	Low	1.06%	4.26%	26.60%	31.91%
			Average	11.70%	14.89%	30.85%	57.45%
			High	6.38%	4.26%	0.00%	10.64%
			Total	19.15%	23.40%	57.45%	100.00%
**Class NCV – Non consistent participants (n = 270)**	**−0.157**	**0.005**	Low	1.48%	4.81%	16.67%	22.96%
			Average	12.96%	18.52%	32.59%	64.07%
			High	2.22%	4.44%	6.30%	12.96%
			Total	16.67%	27.78%	55.56%	100.00%
**Total (n = 364)**	**−0.236**	**< 0.001**	Low	1.37%	4.67%	19.23%	25.27%
			Average	12.64%	17.58%	32.14%	62.36%
			High	3.30%	4.40%	4.67%	12.36%
			Total	17.31%	26.65%	56.04%	100.00%

Nonetheless, there are different intensities between the two Classes: Kendall’s Taub = −0.443 and −0.157 for Class CV and NCV, respectively. This result suggests that volunteers from Class NCV share a less negative opinion concerning the potential impact of humans on the ecological crisis in respect to their counterparts.

### 3.4. Leisure time

About 85% of volunteers state that the pleasure of feeling closer to nature represents a positive feature of participating in the MIPP/InNat initiative, and almost all volunteers (97%) list the promoted activity among their hobbies. Recording insect data appears to be a “social” activity (i.e., carried out with other people): ca. 66% of respondents (240 individuals) state that they involved other people in the activities proposed by MIPP/InNat: among them, siblings have the most relevant share (76%), followed by friends (ca. 55%) and other people (e.g., colleagues) (ca. 49%). In this regard, volunteers from Class CV tend to be keener to engage other people compared to the ones from Class NCV: indeed, the difference between the two classes concerning the tendency to invite other people, despite being very small (ca. 66% vs. 63% for Classes CV and NCV, respectively), results statistically significant (p = 0.024). According to the performed risk analysis (Table 5), in respect to Class NCV, Class CV participants tend to invite others to take part to MIPP/InNat more as highlighted by the odds ratio = 1.715. However, there are no significant differences in inviting siblings or acquaintances whereas there is significant evidence when it comes to inviting friends (odds ratio = 1.642). Moreover, even if the relative risk may not always be clear-cut for inviting people in general and friends in particular (1.182 and 1.355 respectively), Class CV participants result to involve their own network of relations to a greater extent in respect to Class NCV. Additionally, even though values are just above the significance threshold (χ² = 3.80), they increase when Classes are stratified by age and gender. More specifically, Class CV participants are keener to invite their families, especially when considering males (χ² = 23.97; Odds ratio = 5.52) between 31 and 45 years old (χ² = 4.64; Odds ratio = 7.35).

**Table 5:** Risk analysis on the variable “sociability”: comparison of OR and RR between Class CV and Class NCV participants concerning the involvement of other people in the activities proposed by MIPP/InNat. * = significant value for χ², above critical value 3.80, and valid confidence interval, minimum extreme above 1.

	Odds ratio		Relative risk	
Type of invited people	Value	Confidence Interval	Value	Confidence Interval
general* (χ² = 4.11)	1.715	1.01 - 3.37	1.182	1.005 - 2.326
siblings (χ² = 3.43)	1.564	0.97 - 3.21	1.234	0.988 - 2.538
friends* (χ² = 4.15)	1.642	1.01 - 3.22	1.355	1.011 - 2.658
other, e.g., colleagues (χ² = 0.289)	1.123	0.68 - 5.93	1.081	0.773 - 5.709

Furthermore, two out of three volunteers (74.5%) from Class CV tend to collect data during dedicated excursions, while such activity is performed by 56% of the Class NCV. In general, 5.8% of volunteers report attending events organised by MIPP/InNat researchers.

As far as Covid-19 related restrictions are concerned (Table 6), 65.9% out of 94 volunteers of Class CV express to having (at least “Fairly”) suffered from not reporting during the pandemic, while this suffering was experienced by 41% Class NCV.

**Table 6:** Contingent analysis between participants Classes about their affective commitment to the project during Covid-19-related restrictions (χ² = 16.01, p = 0.001; Kendall’s Tau-b = −0.186, p < 0.001).

	Did you suffer from not reporting for the MIPP/InNat project during Covid-19-related restrictions?				
	**Not at all**	**Slightly**	**Fairly**	**Very**	**Total**
**Class CV – Consistent participants (n = 94)**	12.77%	21.28%	39.36%	26.60%	100.00%
**Class NCV – Non-Consistent participants (n = 270)**	22.59%	36.30%	30.37%	10.74%	100.00%
**Total (n = 364)**	20.05%	32.42%	32.69%	14.84%	100.00%

### 3.5. Project’s communication strategies and use of digital technologies

A relevant potential set of enablers for engagement are communication strategies which often rely on digital technologies. Volunteers appear to mostly use the mobile applications: 50% of them affirm to use it very often. Then, volunteers were asked to state how they first knew about MIPP/InNat (Table 7). Most of the volunteers could not remember and were therefore excluded from the analysis, leading to responses only from ca. 25% of the volunteers who took part in the survey, equally distributed between the two classes: 23 out of 94 (24.5%) from Class CV and 68 out of 270 (25.2%) from Class NCV. The most relevant access point to be engaged in MIPP/InNat was the direct face-to-face interaction: 39.6% of volunteers declared to have started gathering insect records after meeting the MIPP/InNat staff. The second most relevant access point to the project was the web (e.g., social networks, webpages, etc.) from which about 29% of volunteers received information, while 18.3% have been engaged by friends and 12.8% read about the project in the press or found some announcements in an attended protected area (data merged into the category “Other”). Considering the two classes of volunteers, a similar pattern to the one described above was found in Class NCV, with face-to-face meetings playing a crucial role in the engagement (ca 41.6%) whereas for Class CV the situation was different. In fact, 33.8% of volunteers from Class CV state to have been engaged after a meeting with MIPP/InNat staff and another 33.8% after having found information online.

**Table 7:** Mode of first contact with MIPP/InNat initiative for the two classes of participants (χ² = 6.69, p = 0.082; Kendall’s Tau-b = −0.116, p = 0.038).

Participants Classes	Direct contact with researchers	Personal contact with friends	Information on the Web	Other	Total
Class CV (n = 71)	33.80%	12.68%	33.80%	19.72%	100.00%
Class NCV (n = 202)	41.58%	20.30%	27.72%	10.40%	100.00%
Total (n = 273)	39.56%	18.32%	29.30%	12.82%	100.00%

Although the significance of this analysis is weak (p = 0.082) it shows that the web is not the most relevant first contact channel for the volunteers to be engaged but it marks a difference between well the two Classes. Indeed, the web plays a sound role for Class CV, as declared by the 33.8% of respondents, though its role decreases when considering Class NCV (27.72%).

## 4. Discussion

The above-reported analyses elaborated data collected through a dedicated web survey whose response rate (22.3%) was satisfactory; as a recent review confirmed that online surveys based on invitation can reach about 44% of response rate on average [50]. In this context, our response rate is lower, but it should be assessed according to two factors, namely the efficiency and the engagement of participants. Firstly, a fully completed questionnaire every four e-mail contacts, calling for voluntary-based participation to the survey, with no reward system, proved quite high efficiency. Secondly, the full list of potential respondents included volunteers that reported a single observation once in ten years; bearing these elements in mind, we can consider our response rate as satisfactory, although to be improved for future research attempts.

### 4.1. Sociodemographic features

Results from the two classes are quite homogeneous, profiling the typical MIPP/InNat volunteer as a mature male with a high level of education, living in a small or medium-sized town in Northern or Central Italy and working as an employee [cf. 51]. A similar result on the age, sex ratio and education level of the participants has been obtained by Larson et al. [52] studying the participants of the Audubon’s Christmas Count, the world’s oldest citizen science project. Even though our data agree with Martellos et al. [53], stating that higher retention rates are evident in older age cases, this pattern does not result statistically significant in our case. The general composition concerning the type of employment accounts only for a few researchers or university professors which is perhaps relatively unexpected, given the efforts made by the project to reach out to schools and universities, especially during the first years. Our analyses detected the existence of a self-selection process which is in line with the discussion on the real potential of citizen science for the democratisation of science [15]: indeed MIPP/InNat mainly attracted and kept engaged participants falling under the demographic of mature male individuals with higher educational attainment [54]. However, literature itself confirms that a recurring element characterising the profile of participants in CS activities is a high level of education, occupation and age [cf. 55,56]. Moreover, as will be further discussed in section 4.2, literature also shows the importance of a rather strong interest in scientific issues, with a high level of cultural consumption of information and entertainment related to scientific research: whether it is simple curiosity or a real passion linked to a hobby, volunteers tend to invest their free time in scientifically meaningful activities. Eventually, literature reports that in many cases there may be a drive to participate in CS projects linked to personal interests in the proposed topic. In the case of projects on biodiversity and nature conservation [57; 58], as the case of MIPP/InNat itself, the driving factor to contribute to the protection of the environment in its various forms must be considered. Given this context, sociodemographic variables and features have been proven to play an indirect crucial role in volunteer engagement [59]: personal values, norms, interest in scientific topics and trust in scientific institutions [60] are also relevant and should be regarded as key factors fostering participants’ engagement.

### 4.2. Interest in scientific topics

It has been proven that environmental CS projects usually attract and engage volunteers who already have a predisposition and curiosity towards nature, as well as enthusiasm in learning new insights about biodiversity and in taking part in nature conservation activities [33,61–64]. No great differences have been found in the two classes concerning interest in scientific topics. In fact, MIPP/InNat volunteers from both classes seem to be truly passionate and fascinated by natural science and entomology and less interested in any utilitaristic perspective on the collected data [e.g., scientific research, photographic interest]. This given, we can assume that volunteers from both classes have been engaged by the project because they are genuinely motivated to do so. In other studies, such as Larson et al. [52], these personal motivations are clearly overcome by the more general ones concerning the importance of supporting species conservation. Thus, our results are in contrast with other studies in which the motivation of participants vary considerably with the acquisition of experience during the project (i.e., from non-consistent to consistent participants): Asingizwe [65] found that initial motivation is connected to curiosity and desire of learning, while it changes, switching to the use of collected data and researchers support with the continuous participation to the project. In this context, Larson et al. [52] found that advancing science and contributing to conservation are two themes that grow accordingly with project participation. The demonstrated interest in biodiversity conservation and monitoring, as well as the one for natural sciences more in general, perfectly fits with the ecocentric values investigated by the survey. In fact, it is no surprise to see a match between the general interest for the environment and the awareness of the ecological crisis and human responsibility, as confirmed in other studies [e.g., 56,66,67].

### 4.3. Ecocentrism and conservation

Being part of an environmental CS project often provides the possibility of acting in close contact with the natural environment thus developing a consequent feeling of attachment and responsibility towards nature and the studied species (or ecosystems) [68,69]. Collecting data for MIPP/InNat seems to enhance such a feeling of being closer to nature and mainly useful for nature conservation. This attitude is well represented in participants belonging to both Classes and it perfectly reflects attitudes largely dominated by ecocentric values. Thus, the observed scenario is one of awareness of the path of the ecological crisis and human responsibility. Although promethean values are less present, this aspect is a clear discriminant between the two Classes and seems to grasp a specific feature of who did participate more constantly across time. In fact, it seems that Class CV volunteers have a more pessimistic view on the human role on the ecological crisis whereas it seems that Class NCV volunteers share a strong belief in the positive impacts of humans on the ongoing ecological and biodiversity crisis. A negative association between promethean and ecocentric values is observed. This can be expected also theoretically [70,71]: indeed, the relevance of values that see humans having a great capacity to solve self-generated environmental problems may appear incompatible with the presence of values related to the ecological crisis, such as ecocentric values, but they still may coexist. While noting these differences, it seems unlikely that the attachment to ecocentric values and the sense of perceived usefulness for environmental protection are decisive elements in ensuring greater consistency across time in participation for reporting activities within the project.

### 4.4. Leisure time

Most volunteers from both Classes list insects recording among their hobbies and state that one of the positive features of participating in MIPP/InNat is the pleasure of feeling closer to nature. This suggests that volunteers probably perceived MIPP/InNat as a good motivation to go for walks and spend time in natural areas. Currently, we suppose that what is observed with the variables connected to leisure time is more probably an effect rather than an element that can explain constancy. Indeed, the link between the affective sphere and reporting constancy becomes clearer when examining the opinion on the period of COVID19 restrictions. While Class CV volunteers suffered from not reporting during the pandemic and are also positively associated with an affective relationship with the project, Class NCV volunteers state a minor suffering and result negatively associated with an affective relationship with the project. Results from Class CV combined with their engagement in open-air leisure activities, in respect to Class NCV, portrays highly committed volunteers who dedicate their free time to reporting contributions. Moreover, we explored sociability, checking if MIPP/InNat provided an opportunity for the engagement of others (e.g., siblings, friends, colleagues): it did in the case of Class CV participants who result to engage their networks more than Class NCV. Although we spotted some statistically significant differences between Class CV and Class NCV (gender and age), it must be taken into account that these are both very similar (see section 4.1) and it is reasonable to think that they do not comprise such different people in terms of world-views (see section 4.2 and 4.3). These are hints that can be explored further by either replicating the survey by applying it to another project, or deepening those hints as hypotheses to be explored in a qualitative way.

### 4.5. Communication and use of digital technologies

The issue of engaging non-professionals or amateurs is very critical in nature monitoring [14,72] and it gained renovated momentum in the last ten years using Information Communication Technologies (ICTs). In fact, ICTs have played a relevant role in the development and increase of projects and participants worldwide [27,73,74]. Indeed, much hope has been placed on the application of ICTs to CS projects in terms of increasing participation [75]; these expectations are reflected in a widespread investment in apps for smartphones and social media between both newer and longer active projects [29]. For example, thanks to ICTs, CS initiatives can encompass small teams or millions of individuals working together towards a shared objective, such as gathering data, analysing it or reporting on findings (“contributory and collaborative projects”, [76]). Moreover, social media and other digital platforms or apps are to date some of the most common strategies to inform and engage potential volunteers: the proliferation of digital technologies is considered a key driver for project success and rise in participation rates [73,77,78]. On the other hand, it has been demonstrated that direct contacts, such as face-to-face interactions, is essential to enhance participants’ motivation [36,79]. Motivating the volunteers through direct personal contacts appears important for keeping them involved: professional researchers involved in public engagement meetings can contribute to promote the CS activity and to create a favourable environment for the development of a constant long-lasting collaboration and network of volunteers. Moreover, a direct engagement through public promotion/workshops can contribute to building commitment in the case of long-lasting projects.

This given, analyses on this dimension only considered volunteers who clearly stated how they first learned about the project. Results partially disconfirm the above-mentioned research results by Cappa et al. [79]: face to face interaction turns out to be relevant but it may need to be supported by online recruiting, or by activities that allow maintaining a constant relationship with the volunteer, which are easier and more rapid if performed online. However, one of the major risks of online communication is that, even though it seems to be effective in recruiting highly motivated volunteers, it also increases the opportunity for self-selection of people already highly engaged in CS or extremely interested in scientific and environmental topics such as biodiversity monitoring. This could be due either to individual motivation (i.e., motivated individuals actively looking for projects to for volunteer) or to algorithmic technologies of search engines and social networks (i.e., algorithms used by any given platform aimed at accommodating user preferences [80]). Even though providing greater inclusiveness by reaching more people who will tend to be highly motivated to stay active in the project, all of this could also result in a challenge that is difficult to address, namely a selective inclusiveness: in the present study case, we have detected a self-selection bias that restricts the project ability to attract volunteers outside the demographic of mature individuals with higher qualifications, as highlighted by Jönsson et al. [54]. Nevertheless, any online platform of a project (primarily social media) aids the recruitment process by engaging volunteers who exhibit a heightened sense of motivation even prior to encountering the project, whether in person or not. In fact, at least theoretically, an online recruiting approach might ensure a consistent and reliable pool of volunteers. Research on recommendation systems in computer sciences consider how to develop new algorithms to increase recruitment rate for projects [81]. As an example, SciStarter (https://scistarter.org/), a portal that matches volunteers with projects, relies on this approach. Given this, algorithmic technologies connected to search and social platforms are certainly a valuable tool for project sustainability, though individual motivation still plays a significant role when compared to other recruitment methods. However, this strategy may address the biases about the age class of participants but it can hardly avoid self-selection biases which seem to dramatically affect participants’ profiles. Considering the two classes of volunteers, these differ on the type of access point stated (i.e., web or face-to-face interactions) and we can interpret such differences as a proxy of the motivational drivers between respondents: it seems plausible to affirm that Class CV volunteers, whose responses were well-balanced between the two main types of access point, have more actively followed and looked for biodiversity contents (e.g., biodiversity monitoring, ecological issues, CS project) compared to volunteers from Class NCV. Notwithstanding the low significance of the contingency analysis reported in Table 6, results from Table 7 may suggest such a relationship and, perhaps, a further research path to be explored in the future.

## 5. Conclusion

A key issue in CS projects is to keep participants actively engaged for a long period. This is particularly relevant in biodiversity projects aimed at the assessment of species conservation status that require a high number of data, ideally largely distributed and covering several years. This given, the present paper explored potential enablers that might keep volunteers active in a project. Based on the long-lasting MIPP/InNat initiative, the analyses explored five areas resuming potential enablers that, according to the literature, are relevant in keeping participants engaged in a biodiversity monitoring project. The survey proposed in 2022 to MIPP/InNat volunteers highlighted the presence of two Classes of volunteers: those most active and “loyal” (Class CV) and those less (Class NCV). As discussed above, the two classes are homogenous, differing significantly only on a few parameters.

Class CV accounts for mature people, predominantly male, with a high level of education. These volunteers are more emotionally committed to MIPP/InNat and show a stronger ecocentric view compared to promethean values. They tend to collect data during dedicated excursions and express more suffering from not reporting during the pandemic. Class CV participants have been involved equally through face-to-face interactions with researchers and online communication.

Class NCV accounts for slightly younger people (thus no statistical significance have been found) in respect to Class CV. They appear to be less emotionally attached to the project, also showing a less ecocentric view and a stronger belief in promethean values. Volunteers from Class NCV tend to collect data during daily routes and felt less regretful about not reporting during the pandemic. They have been mainly involved by face-to-face interactions, with online communication playing a less relevant role.

In general, although MIPP/InNat did not directly involved participants in practices aimed at proactively support biodiversity and the species investigated by the project, as other conservation CS projects may do, e.g., [33,82], it must be noted that volunteers result to be very committed (e.g., they consider the activities promoted by the project as a hobby): this could be possibly due to the self-selection process. Although such a process guarantees a core of motivated volunteers, in turn it can become a bias: as many researchers report, in general CS volunteers tend to be more highly educated and already interested in the topics proposed by a project. This is not negative *per se*, but it represents the outcome of a self-selection process that is difficult to overcome. Also, MIPP/InNat has a limited ability to attract participants outside the above-outlined demographic. Coherently, the study highlights the importance of personal networks of volunteers as potential trigger for volunteer recruitment. Class CV participants are more prone to invite other people to join the project compared to Class NCV participants: they invite people with whom they are in close relationship (siblings and friends), that perhaps share the same interests (e.g., open air activities, biology or even insects). In conclusion, the present study provides a detailed account of MIPP/InNat volunteers, also prompting a re- evaluation of the position of online recruitment. The above-mentioned results are to be considered a preliminary approach to the sociological aspects concerning MIPP/InNat as a case study: further examination employing other research methods, such as in-depth interviews, is thus required to gain more detailed insights into additional factors that could facilitate more comprehensive and successful campaigns for CS project, especially in terms of volunteer retention.
